# Promoters for Improved Adhesion Strength between Addition-Cured Liquid Silicone Rubber and Low-Melting-Point Thermoplastic Polyurethanes

**DOI:** 10.3390/ma15030991

**Published:** 2022-01-27

**Authors:** Jia-Kai Wu, Kai-Wen Zheng, Xing-Cheng Nie, Huang-Rong Ge, Qiong-Yan Wang, Jun-Ting Xu

**Affiliations:** 1 MOE Key Laboratory of Macromolecular Synthesis and Functionalization, Department of Polymer Science & Engineering, Zhejiang University, Hangzhou 310027, China; 11429008@zju.edu.cn (J.-K.W.); 15117965820@163.com (K.-W.Z.); xujt@zju.edu.cn (J.-T.X.); 2 Research and Development Center, Zhejiang Sucon Silicone Co., Ltd., Shaoxing 312088, China; sxyxnxc@163.com (X.-C.N.); ghuangrong@163.com (H.-R.G.)

**Keywords:** self-adhesion, liquid silicone rubber, thermoplastic polyurethanes, cohesive failure

## Abstract

A polydimethylsiloxane armed with epoxy, alkoxy and acrylate groups was synthesized from silanol terminated-PDMS and epoxy and acrylate groups functionalized silane coupling agents, and utilized as the adhesion promoter (AP) to prepare addition-cured liquid silicone rubber that exhibited self-adhesion ability (SA-LSR) with biocompatible thermoplastic polyurethanes (TPU) sheets. The structural characteristics of AP were characterized by Fourier transform infrared (FTIR) spectroscopy, which demonstrated the strong adhesion to polyester-based TPU sheets due to a sufficient amount of acrylate groups, epoxy groups and silanol groups obtained by the hydrolysis of alkoxy groups. In detail, the peel-off strength of SA-LSR and TPU joints reached up to 7.63 N mm^−1^ after the optimization of adhesion promoter including type and content, and curing condition including time and temperature. The cohesive failure was achieved during the sample breakage process. Moreover, the SA-LSR showed a good storage stability under proper storage conditions. This design strategy provided the feasibility to combine the advantages of addition-cured liquid silicone rubber and plastics with low melting points, promoting the potential application range of those silicone-based materials.

## 1. Introduction

The attachment of “organic” groups to the Si–O bond “inorganic” backbone endows silicones with a distinct combination of properties including heat resistance, chemical stability, electrical insulating, abrasion resistance, weatherability and excellent biocompatibility, hence facilitating a wide range of application of silicones in aerospace, automobile, construction, electronic and electrical, and medical industries, etc. [1,2,3,4]. The combination of silicones with other polymer materials provides an attractive route to develop new materials with desirable properties [5,6]. Recently, the development of addition-cured liquid silicone rubber (LSR) with no curative by-products and easy automatic process has grown rapidly due to the large demand for “healthier” silicones in biomedical industries [7,8,9]. Thermoplastic polyurethane (TPU), showing good mechanical properties, aging resistance and abrasion resistance, is one of the most important polymer materials that has been widely used in biomedical field [10,11,12]. Materials that combine the advantages of LSR and TPU spur huge economic interests in the fields of smart wearable devices, mobile communications, medical beauty, etc. [13,14,15].

However, LSR cured through the hydrosilylation reaction between Si-H bonds and vinyl groups in different types of polydimethylsiloxane (PDMS), which leads to low chemical reactivity and surface energy, hence weak adhesion to non-silicone-based substrates [1]. This deteriorates the compatibility between silicones and other materials to prepare high-performance composite materials [16]. In recent years, enormous efforts have been devoted to enhance the adhesion strength of LSR on various substances surfaces [17,18,19,20]. The surface modification is an effective method to improve the adhesion strength between LSR and other materials by creating new reactive groups on the substrate surface [21,22,23]. Seitz et al. reported an atmospheric pressure plasma jet and Pyrosils^®^ flame method to modify the surface of polyetheretherketone (PEEK) [21]. The yielded oxygen or nitrogen containing reactivity groups and Pyrosils^®^ layer on the PEEK surface led to a significant improvement in adhesion with LSR. The composite PEEK and LSR joint showed cohesive failure during 90° peel-off test. The use of primers is another method to achieve effective adhesion between LSR and substrates. Primers, consisting of silane coupling agent, titanate, silicone resin or methacrylate, show good wettability to most substrates, film-forming properties as well as good chemical compatibility with silicones, which are conducive to increasing the interfacial adhesion ability [24,25,26]. Chang et al. adopted a commercial primer containing active ingredients of polymethyl methacrylate and polyorganosiloxane (Tokuyama Sofreliner T) to treat the polyurethane (PU) surface before bonding with silicone rubber to enhance the adhesion strength [24]. The resultant addition-cured silicone A-2000 (Factor II, Inc., Lakeside, AZ, USA) and PU assembly showed pure cohesive failure during T-peel test. Both aforementioned surface modification and use of primers can effectively improve the adhesion ability between LSR and various substrates, however, the two methods have time-consuming and complicated process issues.

Recently, self-adhesive liquid silicone rubber (SA-LSR) is attracting extensive attention, which contains functional siloxane oligomers (e.g., epoxy, alkoxy, methacryloxy groups) serving as effective adhesion promoters toward various substrates in the curing process [27,28,29,30]. Siloxane oligomers containing boron and epoxy groups were synthesized by Pan et al. and utilized as adhesion promoter to promote the adhesion strength of LSR to polyphthalamide (PPA) and LSR to copper (Cu) plates, respectively [27]. The prepared LSR exhibited good self-adhesive properties with PPA and Cu plates. The shear strength of the LSR/PPA and LSR/Cu joints reached up to 3.16 MPa and 1.69 MPa, respectively, which were 3.92 times and 2.57 times higher than those of LSR without adhesion promoter. Tsai et al. reported a hydroxyl-functionalized polydimethylsiloxane-block-acrylate prepolymer copolymer that served as an adhesion promoter to reconstruct the surface of liquid silicone rubber and improve the adhesion between LSR and PU [31]. The enrichment of acrylate prepolymer blocks on the LSR surface enhanced the peel strength between LSR and PU from 0 to 10.8 N cm^−2^. However, those common adhesion promoters of modified SA-LSR show unsatisfactory adhesion strength and durability towards thermoplastic substrates, especially when they are bonded to low melting-point polymer substrates that cannot be cured at high temperature. Therefore, a new strategy for the synthesis of adhesion promoter, which can endow LSR with strong adhesion to low melting-point TPU substrate is urgently needed.

The LSR/TPU assembly is urgently needed in smart wearable devices and medical beauty fields [7,13]. To this end, addition-cured LSR with strong self-adhesion strength towards polyester-based TPU sheet was engineered, in which polydimethylsiloxane functionalized with epoxy, alkoxy and acrylate groups was utilized as the adhesion promoter. The adhesion promoter was synthesized via a condensation reaction between silanol groups in silanol-terminated polydimethylsiloxane and alkoxy groups in functionalized silane coupling agents. 2-(3,4-Epoxycyclohexyl) ethyl trimethoxysilane containing highly reactive epoxy groups and 3-methacryloxypropyl methyl dimethoxysilane containing acrylate groups that are similar to polyester structure were carefully selected as the functionalized silane coupling agents. The polydimethylsiloxane backbones attributed good compatibility to adhesion promoter with LSR matrix while the reactive epoxy, alkoxy and acrylate groups provided the adhesion promoter strong adhesion ability to TPU substrate. The structural characteristics of adhesion promoter was characterized by Fourier transform infrared spectroscopy and gel permeation chromatography, and the adhesion ability of SA-LSR to TPU substrate were systematically investigated via T-peel test. The curing and storage conditions of SA-LSR were evaluated in detail.

## 2. Materials and Methods

### 2.1. Materials

3-Methacryloxypropyl methyl dimethoxysilane (MPMDS, >98.0%), 2-(3,4-Epoxycyclohexyl) ethyl trimethoxysilane (ECETS, >97.0%), Tetrabutyl titanate (>99.0%) and 3,5-dimethyl-1-hexyn-3-ol (>98%) were all obtained from Aladdin, Shanghai, China. Silanol-terminated polydimethylsiloxane (PDMS-OH) with different viscosities were purchased from Sigma-Aldrich (St. Louis, MO, USA) (viscosities of ~25 cSt and ~65 cSt) and Gelest, Inc (Morrisville, PA, USA) (viscosity of ~35–45 cSt) and the PDMS-OH were marked as PDMS-OH-1, PDMS-OH-2 and PDMS-OH-3 as the increasement of the viscosities. Vinyl-terminated polydimethylsiloxane (PDMS-Vi, PDMS-Vi-1: vinyl content = 0.06 wt.%, PDMS-Vi-2: vinyl content = 0.14 wt.%), poly(methyl-hydro-co-dimethylsiloxane) (PMHS, hydrogen content = 0.18 wt.%) and hydrophobic fumed silica were provided by Zhejiang Sucon Silicone Co., Ltd., Shaoxing, China. The divinyltetramethyldisiloxane platinum catalyst (platinum content = 0.5 wt.%) was acquired from Heraeus Materials Technology Shanghai Ltd. (Shanghai, China). Polyester-based thermoplastic polyurethanes (TPU) with a melting point of 431 K was gained from DingZing Advanced Materials Inc., Kaohsiung, Taiwan.

### 2.2. Synthesis of the Epoxy, Alkoxy and Acrylate Groups Modified Polydimethylsiloxane

**Figure 1:**
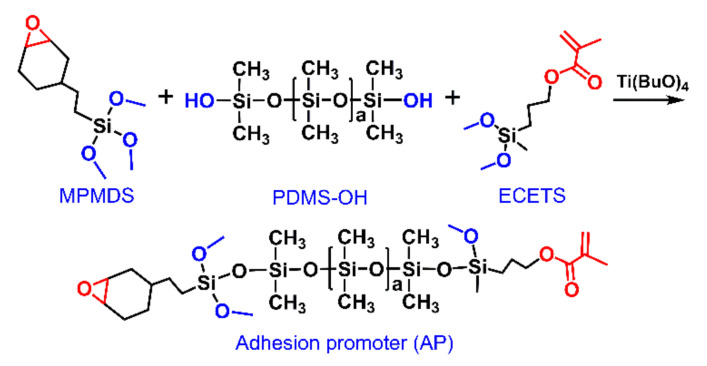
Schematic diagram for the synthesis of epoxy, alkoxy and acrylate groups modified PDMS adhesion promoter.

The adhesion promoter epoxy, alkoxy and acrylate groups modified PDMS was synthesized via a condensation reaction between silanol groups and alkoxy groups as shown in Figure 1. In detail, PDMS-OH (50.0 g) and tetrabutyl titanate (0.10 g) were added into a four-neck flask, which was equipped with a mechanical stirrer, thermometer and reflux and nitrogen-blow devices. The flask was backfilled with nitrogen and placed into the oil bath, stirred and thermostated at 348 K for 0.5 h. Subsequently, the stoichiometric amount of MPMDS and ECETS mixtures (1:1, mol/mol) were added into the flask dropwise within 10 min and stirred for another 1 h. After cooling down to room temperature, activated carbon (3.5 g) was added into the mixtures to absorb the residual titanate catalyst, and was then removed by negative pressure filtration. The unreacted molecules and yielded alcohols were removed by vacuum distillation to yield the desired adhesion promoter as liquid. The adhesion promoter is designated as AP-1, AP-2 and AP-3, corresponding to three types of PDMS-OHs (i.e., PDMS-OH-X, X = 1, 2, 3).

### 2.3. Preparation of SA-LSR Samples

PDMS-Vi and hydrophobic fumed silica were mixed in an internal mixer for 2 h followed by a heat treatment at 423 K for 1 h to obtain uniform silicone rubber blends. When cooling down to ambient temperature, the obtained silicone rubber blends were mixed with PHMS, platinum catalyst, 3,5-dimethyl-1-hexyn-3-ol and AP-X according to a certain ratio in a planetary mixer. The silicone rubber was degassed for 2 min under a 266.6 Pa vacuum. As-prepared self-adhesive silicone rubbers with the addition of three AP-X were marked as LSR-AP-1, LSR-AP-2, LSR-AP-3, respectively. On the other hands, five LSR-AP-2 samples with different contents of AP-2 were prepared by adjusting the weight ratio of AP-2 to LSR was 0.5, 1, 1.5, 2 and 3, respectively. The detailed compositions of SA-LSR was shown in Table 1.

### 2.4. Characterization

The chemical structure of AP-X was characterized by Fourier transform infrared spectroscopy (FT-IR, Nicolet iS10, Thermofisher Scientific, Waltham, MA, USA). The samples were prepared by coating a thin film of AP on the KBr slice (25 mm × 2 mm). The relative molecular mass (*M_n_*), relative molar mass (*M_w_*) and molar-mass dispersity of PDMS-OH and AP were tested by a gel permeation chromatography (GPC, Waters 1515, Milford, MA, USA) equipped with a Waters 2414 refractive-index detector and using tetrahydrofuran (sample concentration 0.1 wt.%, flow rate 1.0 mL min^−1^, 303 K) as an eluent. The viscosity of SA-LSR was measured with a viscometer (DV2T, Brookfield, WI, USA) at 298 K. The mechanical properties of SA-LSR samples were measured using a universal testing machine (AI-7000S, Gotech, Taiwan), which was carried out at a stretching rate of 20 mm min^−1^ at 298 K. SA-LSR samples were cured by a hydrosilylation reaction at 393 K for 30 min followed at 298 K (50% RH–70% RH) for another 24 h. The SA-LSR samples were punched out into dumbbell shapes (according to GB/T 528) before tensile tests. The mean values were derived from 5 samples of each measurement (deviation should less than 10%). The microscopic morphologies at the breaking area of LSR/TPU and SA-LSR/TPU joints were visualized by field emission scanning electron microscopy (FESEM, S4800, Hitachi, Japan) at voltages of 3 kV and were sputtered with platinum beforehand. The fatigue test was performed by stretching a 100 mm SA-LSR/AP-2 and TPU joint to 200 mm (100% elongation) at a speed of 50 mm min^−1^, followed by removing the applied force to restore the component as one cycle.

### 2.5. Adhesion Strength Test

**Figure 2:**
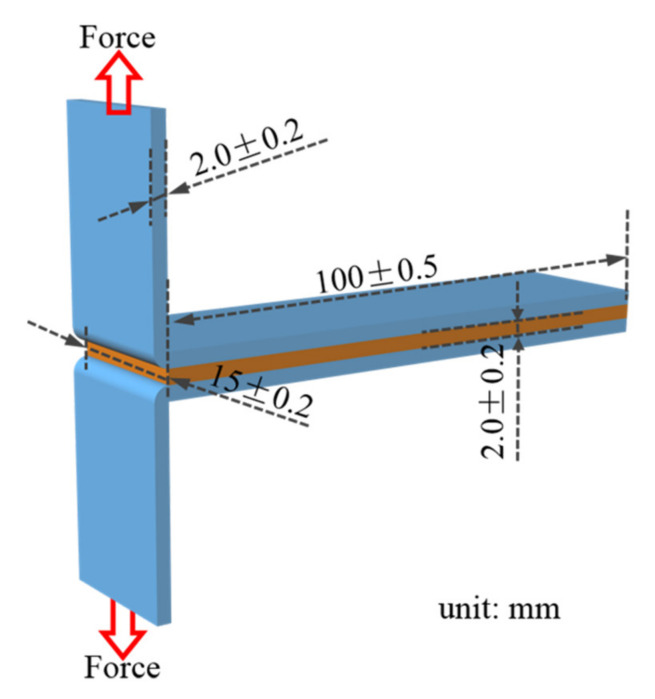
Schematic diagram of SA-LSR/TPU samples in T-peel test.

The peel-off strength is generally regarded as the key parameter to evaluate the adhesion performance of the flexible-to-flexible interfaces, which can reduce the flexible deformation of the material during the pulling process [32]. Therefore, T-peel tests were performed by a universal testing machine (AI-7000S, Gotech Taiwan) to evaluate the adhesion performance of SA-LSR to TPU sheets (Figure 2). TPU sheets were scrubbed with isopropanol and dried at 308 K for 30 min. Quantitative SA-LSR was sandwiched between two TPU sheets under a 0.2 MPa pressure and cured at 393 K for 20 min, and as-prepared SA-LSR/TPU was kept at room temperature (RT) for 24 h. The effective bonding area (overlapping area between SA-LSR and TPU) of the samples was 15 × 100 mm^2^, and the thickness was 2 mm. The peel-off strength between SA-LSR and TPU sheets was determined by the following equation:

*τ_p_* = *F_p_*/*w* (1)

where *τ_p_* is the peel-off strength between SA-LSR and TPU sheets (N mm^−1^), *F_p_* represents the averaged force (N) during the T-peel tests where TPU sheet is peeled off from SA-LSR with the total length of 75 mm (the initial area of 25 mm is not taken into account), *w* is the width of the samples (15 ± 0.2 mm).

## 3. Results

### 3.1. Characterizations of AP

**Figure 3:**
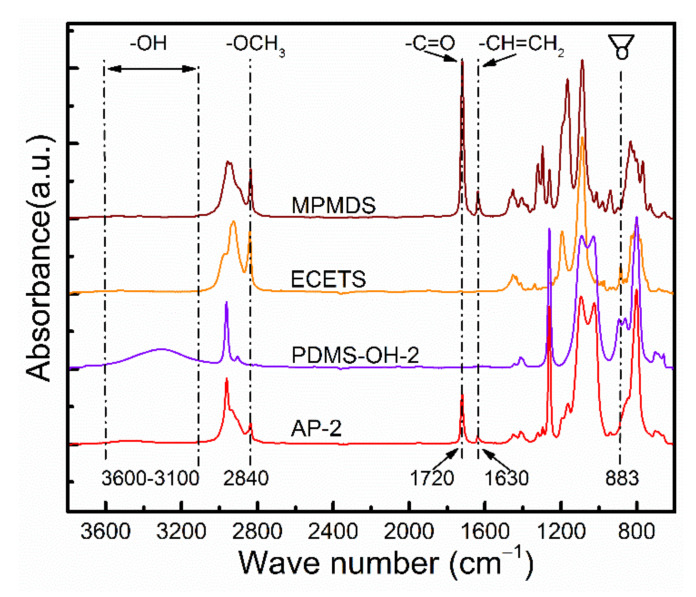
FT-IR spectra of MPMDS, ECETS, PDMS-OH-2 and AP-2.

ECETS armed with highly reactive epoxy groups and MPMDS containing acrylate group, which possessed a relatively similar structure to polyester (in TPU) were selected as functionalized silane coupling agents to synthesize the adhesion promoter. Additionally, the chemical structure of AP-2 was identified by FT-IR as shown in Figure 3. The broad strong peak between 3200–3600 cm^−1^ was assigned to the stretching vibration of -OH groups in PDMS-OH-2 [33]. The characteristic absorption peaks at 2840 cm^−1^, 1720 cm^−1^ and 1630 cm^−1^ were ascribed to the stretching vibration of -Si-OCH_3_ groups, >C=O groups and –CH=CH_2_ groups in MPMDS, respectively [34]. The absorption peak of epoxy groups in ECETS appeared in 883 cm^−1^ [35]. The disappearance of absorption peak of -OH groups accompanied by the weakening of absorption intensity of -Si-OCH_3_ groups in AP-2 indicated the successful condensation reaction between Si-OH (in PDMS-OH) and alkoxy groups (in MPMDS and ECETS). The new peaks of >C=O groups, –CH=CH_2_ groups and epoxy groups in AP-2 demonstrated that the PDMS-OH-2 was modified with MPMDS and ECETS successfully. After the condensation reaction, the increased relative molecular mass and relative molar mass of AP-2 compared with its corresponding raw material PDMS-OH-2 further indicated that the epoxy groups, alkoxy groups and acrylate groups functionalized AP-2 was successfully synthesized (Table 2), while similar molar-mass dispersity between PDMS-OH-2 and AP-2 manifested that no self-condensation occurred in PDMS-OH or silane coupling agents. However, a small amount of AP-2 terminated with dual-ECETS or dual-MPMDS groups was inevitable in this condensation reaction. The similar changes in FT-IR spectra (Supplementary Materials Figure S1) and molecular weights were also observed in AP-1 and AP-3 synthesized from PDMS-OH-1 and PDMS-OH-3, revealing a simple synthesis approach for the modification of PDMS with epoxy, alkoxy and acrylate groups.

### 3.2. Mechanical Properties of SA-LSR

**Figure 4:**
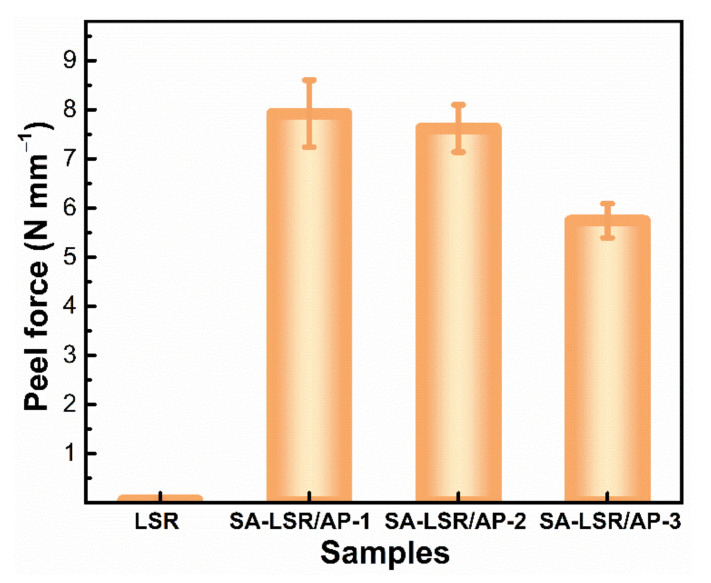
The effect of three adhesion promoters on the peel strength between LSR/SA-LSR and TPU sheets (LSR without any adhesion promoters, SA-LSR with 2.0 wt.% AP-X).

The effect of three adhesion promoters on the peel forces between SA-LSR and TPU sheets was investigated by T-peel test and shown in Figure 4. The original LSR was weakly adhesive to TPU, that is, the TPU could be easily peeled off from the LSR sheet by applying only a small force (*τ_p_* < 0.1 N mm^−1^). This is mainly due to the weak interactions between TPU and LSR for the low surface energy and the lack of reaction groups in original LSR sheet. All three adhesion promoters exhibited significant improvement in the adhesive ability of LSR to TPU. In detail, the peel-off strength between three SA-LSR and TPU sheets were approaching 7.92 N mm^−1^, 7.63 N mm^−1^ and 5.37 N mm^−1^ by adding 2 wt.% AP-1, AP-2, and AP-3, respectively. Moreover, SA-LSR with 2 wt.% addition of AP-1 or AP-2 showed cohesive failure at the peeling interfaces. The strong adhesion was attributable to the abundant reactive groups including epoxy groups, alkoxy groups and acrylate groups in the adhesion promoters which formed a large number of chemical and hydrogen bonds between SA-LSR and TPU sheets at the interface and high temperature. Besides, the vinyl groups in adhesion promoters could be tightly connected with LSR matrix via the hydrosilylation reaction during curing process. Regardless of the increased adhesion ability for all three SA-LSR, AP-3 modified SA-LSR showed less bonding effect to TPU (about 60% area of cohesive failure) than those of AP-1 and AP-2 modified SA-LSR due to the less reactive groups in AP-3.

**Figure 5:**
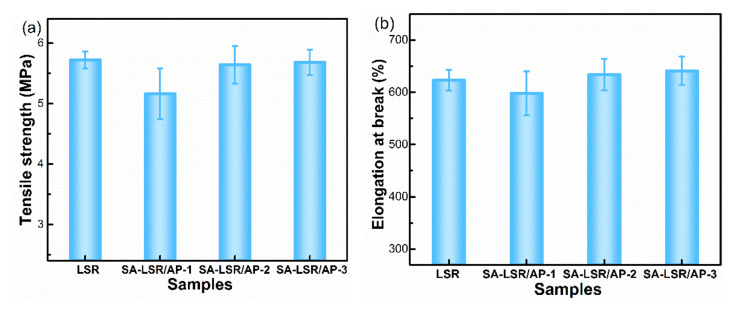
The effect of the three adhesion promoters on (**a**) tensile strength and (**b**) elongation at break of LSR and SA-LSR (LSR without any adhesion promoters, SA-LSR with 2.0 wt.% AP-X).

Figure 5 presented the effect of three adhesion promoters on the mechanical properties of SA-LSR. The tensile strength and elongation at break of LSR without any adhesion promoter was 5.72 MPa and 623%, respectively, and those of SA-LSR with 2 wt.% AP-2 or AP-3 showed little difference compared with the original SA-LSR. After incorporating 2 wt.% AP-1, however, the tensile strength and elongation at break of SA-LSR decreased by 9.8% and 4.1%. The large number of polar groups in the AP-1 formed strong interactions with SiO2 in SA-LSR and resulted in a substantial increase in the viscosity of SA-LSR (Supplementary Materials Figure S2) [36]. The rapidly increased viscosity made it difficult for the adhesion promoter AP-1 to mix uniformly in SA-LSR, while the strong polarity of AP-1 caused poor compatibility with LSR matrix, both of which led to the slightly reduced mechanical properties of the SA-LSR. Considering the mechanical properties and adhesion ability of SA-LSR, adhesion promoter AP-2 with proper polydimethylsiloxane segments and polar reactive groups was selected for the subsequent research.

### 3.3. Adhesion Performance of SA-LSR

**Figure 6:**
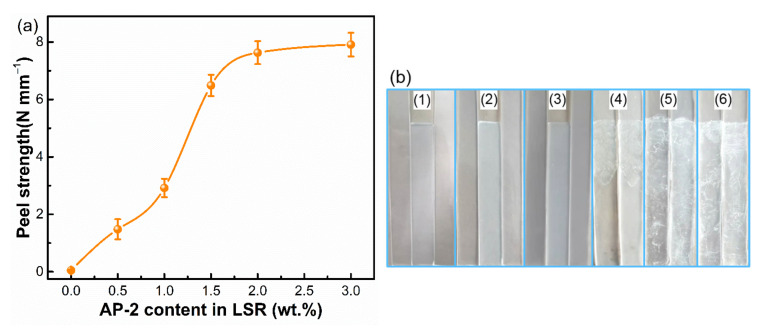
(**a**) Effect of AP-2 content on the peel strength between SA-LSR and TPU sheets, (**b**) optical photographs of the LSR/TPU and SA-LSR/TPU joints after peel tests. AP-2 content in the LSR was 0, 0.5, 1, 1.5, 2, 3 wt.% from (1) to (6), respectively.

The effect of AP-2 content on the peel strength between SA-LSR and TPU sheets was investigated. The LSR with no adhesion promoter showed weak adhesive ability to TPU sheets and could be easily and completely peeled off from TPU sheets (adhesive failures, Figure 6). By increasing the AP-2 content from 0.5 wt.% to 2.0 wt.%, the peel strength between SA-LSR and TPU sheets increased from 1.48 N mm^−1^ to 7.63 N mm^−1^, and the adhesive ability of SA-LSR improved significantly as the AP-2 content exceeded 1 wt.%. As the AP-2 content in the samples exceeded 1 wt.%, the SA-LSR and TPU joint exhibited adhesive and cohesive failures after the peeling test. In particular, the cohesive failure on a macroscale was observed at the interface of SA-LSR and TPU joint after the peeling test when the AP-2 content in SA-LSR reached up to 2 wt.% (Figure 6b). The peel strength between the SA-LSR and TPU sheets was enhanced and smoothed as the AP-2 content continued to increase to 3 wt.%. Besides, the LSR/TPU sample showed a uniform smooth surface micromorphology at the breaking area, while the SA-LSR/TPU sample presented a rough surface micromorphology with a slight aggregation of silica particles (Supplementary Materials Figure S3). This significant enhancement of peel strength was mainly attributed to the interface enrichment of AP-2, that is, the polar AP-2 in the SA-LSR migrated to the interface with the polar groups orientated toward polar TPU side during the curing process [37]. Additionally, the enrichment of AP-2 on the interface endowed SA-LSR with sufficient adhesive ability to TPU sheets. The concentration of adhesion promoter at the interface increased with increasing the AP-2 content in SA-LSR, which was consistent with the monotonically improved peel strength between SA-LSR and TPU sheets. When the adhesion promoter in the SA-LSR exceeded 2 wt.%, the content of AP-2 on the surface was kept almost unchanged due to the space constraints, hence the unobvious improvement of adhesive ability. Therefore, the 2 wt.% addition of AP-2 to SA-LSR is the optimal content to provide sufficient adhesion to bind TPU sheets.

**Figure 7:**
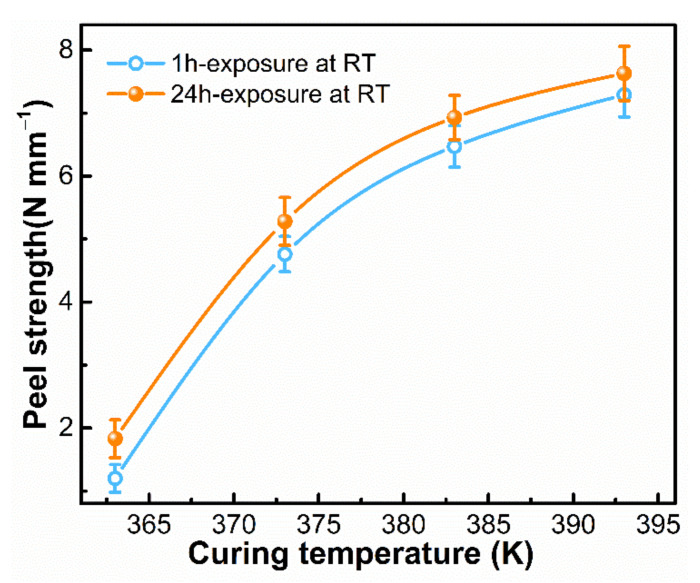
Effect of curing temperature and exposure time on peel strength between SA-LSR (with 2 wt.% AP-2) and TPU sheets, curing time: 30 min.

The effect of curing condition on peel strength between SA-LSR and TPU sheets was investigated and shown in Figure 7. With increasing the curing temperature from 363 K to 393 K, the peel strength increased rapidly from 1.83 N mm^−1^ to 7.63 N mm^−1^. The enhancement in peel strength was mainly ascribed to the increased reactivity of epoxy groups in adhesion promoter with increasing the curing temperature, which facilitated the chemical bond formation with amino groups in TPU. Moreover, the thermal movement of polyester soft segments in TPU was easier at higher temperature, resulting in the increased molecular interactions between polyester chains and acrylate groups in AP-2. The curing temperature did not increase further by considering that the melting point of selected TPU is about 423 K. In particular, the peel strength of the cured SA-LSR and TPU sheets tested after 24 h-exposure at room temperature was higher than that tested immediately after cooled down to room temperature for around 1 h. The hydrolysis of alkoxy groups in AP-2 occurred slowly with the moisture in atmosphere hence generating a large amount of silanol groups [38]. The yielded silanol groups were highly reactive groups that were responsible for the chemical bond formation with TPU sheets and improved the peel strength.

**Figure 8:**
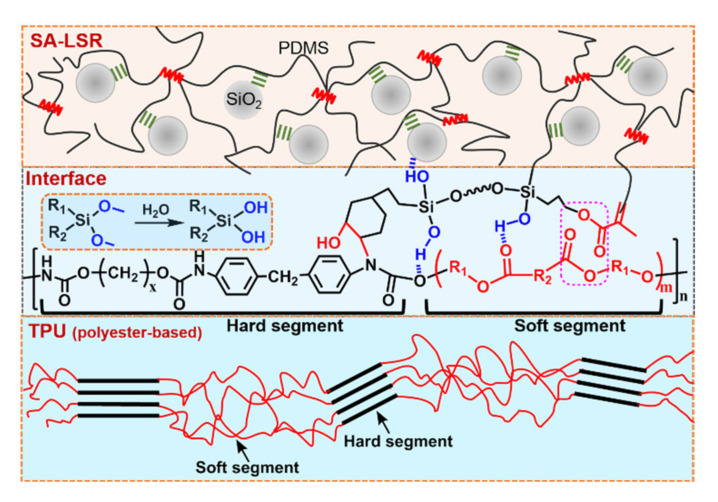
Schematic diagram of the adhesion mechanism of AP-2 modified SA-LSR adhesive to TPU sheets.

On the basis of the result, an adhesion mechanism model of the AP-2 modified SA-LSR bonding to polyester-based TPU sheets was proposed and depicted in Figure 8. The AP-2 migrates and enriches at the surface of SA-LSR and TPU sheets with high surface energy, and the SA-LSR forms chemical bonds with TPU sheets at the interface via the reaction of epoxy and amine groups. Besides, the increased thermal motion of polyester soft segments in TPU leads to strong molecular interaction with acrylate groups in AP-2 at high temperature. The obtained silanol groups further enhances the adhesive ability of SA-LSR to TPU sheets. Additionally, the vinyl groups in AP-2 make the adhesion promoter tightly interact with SA-LSR matrix. Thus, SA-LSR/AP-2 exhibited good adhesion strength (peel force 7.63 N mm^−1^) to TPU sheets, comparable to those of commercialized LSR BQ-7640A/B (peel force 6.20 N mm^−1^, Betterly New Materials Co., Ltd., Dongguan, China) and Silopren* LSR 47 × 9 (peel force 8.60 N mm^−1^, Momentive Performance Materials Inc, Waterford, NY, USA).

**Figure 9:**
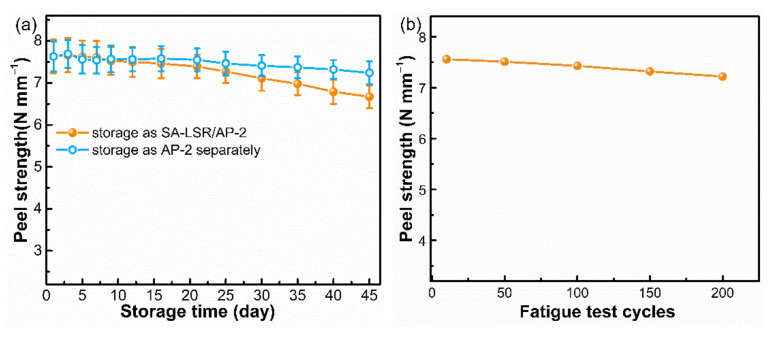
(**a**) Dependence of the peel strength on storage time and condition between SA-LSR (with 2 wt.% AP-2) and TPU sheets at 298 K. (**b**) The effect of fatigue test cycles on the peel strength of SA-LSR/AP-2 and TPU joint.

The long-term storage stability is critical to the commercial application of SA-LSR and evaluated by two methods [30]. The first is that SA-LSR and AP-2 were mixed and stored together in a polypropylene container, while another method is that the SA-LSR and AP-2 were stored separately and AP-2 was added to the SA-LSR before the curing procedure. As shown in Figure 9, SA-LSR prepared by two methods presented good adhesion strength in the first four weeks. The SA-LSR with AP-2 and the LSR being stored separately maintained good bonding ability to TPU sheets even after six weeks of storage, confirming excellent storage stability. However, the SA-LSR with pre-added AP-2 showed a 12.5% reduction of adhesion strength after the total storage period of six weeks. Moreover, the SA-LSR/AP-2 and TPU joint showed a stable peel strength and cohesive failure was observed after 200 cycles in fatigue test, indicating good fatigue resistance of the adhesion assembly (Figure 9b).

## 4. Conclusions

In summary, the epoxy, alkoxy and acrylate groups functionalized PDMS was synthesized via a condensation reaction between silanol and alkoxy groups and utilized as an adhesion promoter to improve the adhesion ability of SA-LSR to TPU sheets. The epoxy groups provided chemical cross-linking structure while the acrylate groups offered strong molecular interaction between SA-LSR and TPU sheets, respectively. Silanol groups yielded by the hydrolysis of alkoxy groups further enhanced the adhesion between SA-LSR and TPU sheets. By incorporating 2.0 wt.% AP-2 and curing at 393 K for 30 min followed at 298 K for another 24 h, as-prepared SA-LSR exhibited the cohesive failure during the separation between SA-LSR and TPU joint, leading to the peel strength reach up to 7.63 N mm^−1^. Moreover, the SA-LSR showed good storage stability under proper storage conditions. This strategy easily combined the advantages of addition-cured liquid silicone rubber and plastics with low melting points, showing a good potential adaptability of liquid silicone rubbers to enhance adhesion ability, but also possessing good application potentials in the fields of smart wearable devices and medical beauty, etc.

## Figures and Tables

**Table 1:** Compositions of SA-LSR samples.

Compositions	Content (g)
PDMS-Vi-1	50.0
PDMS-Vi-2	90.0
PHMS	3.8
Hydrophobic fumed silica	20.0
Platinum catalyst	0.2
3,5-dimethyl-1-hexyn-3-ol	0.3
AP-2	0–5.0

**Table 2:** Relative molecular mass (*M_n_*), relative molar mass (*M_w_*) and molar-mass dispersity of PDMS-OH and AP.

Samples	*M_n_*	*M_w_*	*M_w_/M_n_*	OH (wt.%) ^a^
PDMS-OH-1	565	898	1.59	6.02
PDMS-OH-2	1028	1452	1.41	3.31
PDMS-OH-3	2205	3321	1.51	1.54
AP-1	1205	1962	1.63	/
AP-2	1605	2392	1.49	/
AP-3	2698	4187	1.55	/

^a^ The -OH contents of PDMS-OH were calculated as: 34/*M_n_* × 100%.

## Data Availability

Not applicable.

## Supplementary Materials

The following supporting information can be downloaded at: https://www.mdpi.com/article/10.3390/ma15030991/s1, Figure S1. FT-IR spectra of PDMS-OH-1, PDMS-OH-3, AP-1 and AP-3; Figure S2. The effect of the three adhesion promoters (AP) on the viscosity of SA-LSR.; Figure S3. The optical photograph and SEM surface morphology at the breaking area of the LSR/TPU samples.
