# Supplementary material for: Promoters for Improved Adhesion Strength between Addition-Cured Liquid Silicone Rubber and Low-Melting-Point Thermoplastic Polyurethanes
Source: Materials (Basel). 2022 Jan 27;15(3):991. doi: 10.3390/ma15030991 (PMC8838879; doi:10.3390/ma15030991)
Supplement: Supplementary file 1 [file materials-15-00991-s001.zip › materials-1559233-supplementary.pdf]

Supplementary Materials

Promoters for Improved Adhesion Strength between Addition-Cured Liquid Silicone Rubber and Low-Melting-Point Thermoplastic Polyurethanes

Jia-Kai Wu [1,2], Kai-Wen Zheng [1], Xing-Cheng Nie [2], Huang-Rong Ge [2], Qiong-Yan Wang [2,*] and Jun-Ting Xu [1]

[1] MOE Key Laboratory of Macromolecular Synthesis and Functionalization, Department of Polymer Science & Engineering, Zhejiang University, Hangzhou 310027, China; 11429008@zju.edu.cn (J.K.W.); 15117965820@163.com (K.W.Z.); xujt@zju.edu.cn (J.T.X.)

[2] Research and Development Center, Zhejiang Sucon Silicone Co., Ltd., Shaoxing 312088, China; sxyxnxc@163.com (X.C.N.); ghuangrong@163.com (H.R.G.)

* Correspondence: wqy2040@163.com

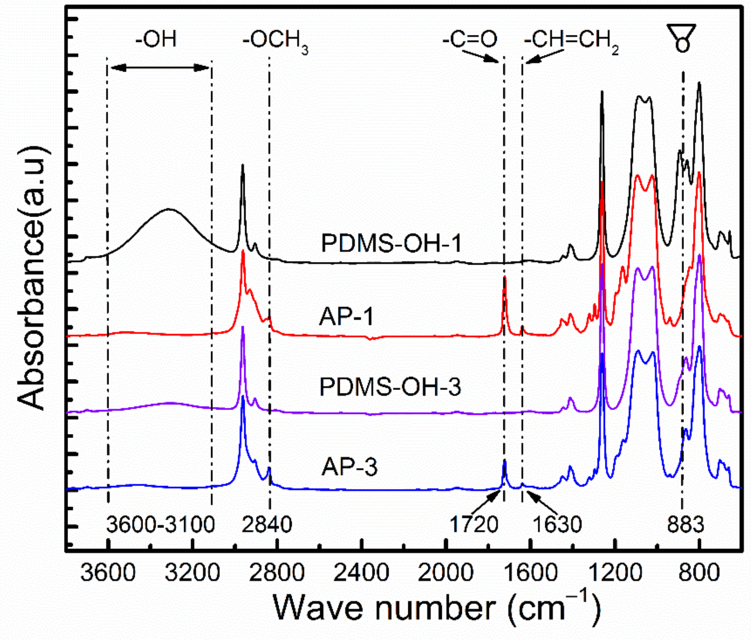

Figure S1. FT-IR spectra of PDMS-OH-1, PDMS-OH-3, AP-1 and AP-3.

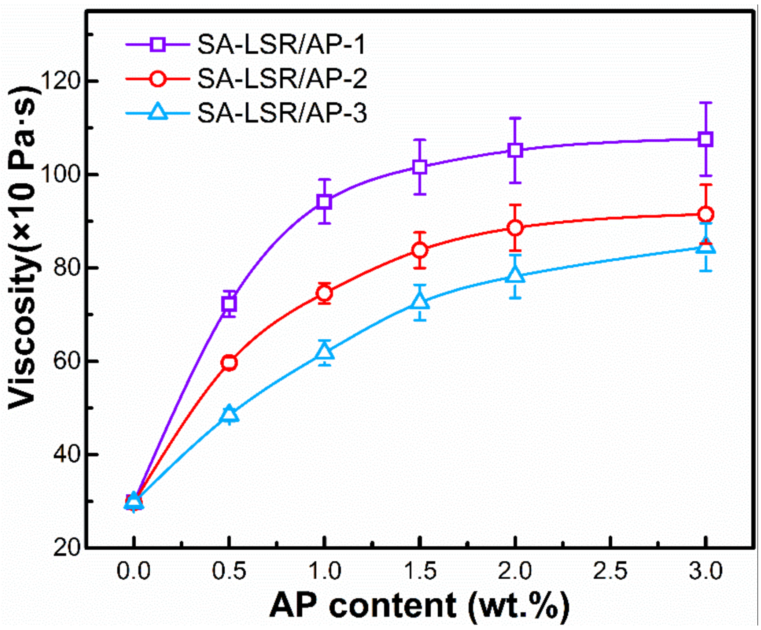

Figure S2. The effect of the three adhesion promoters (AP) on the viscosity of SA-LSR.

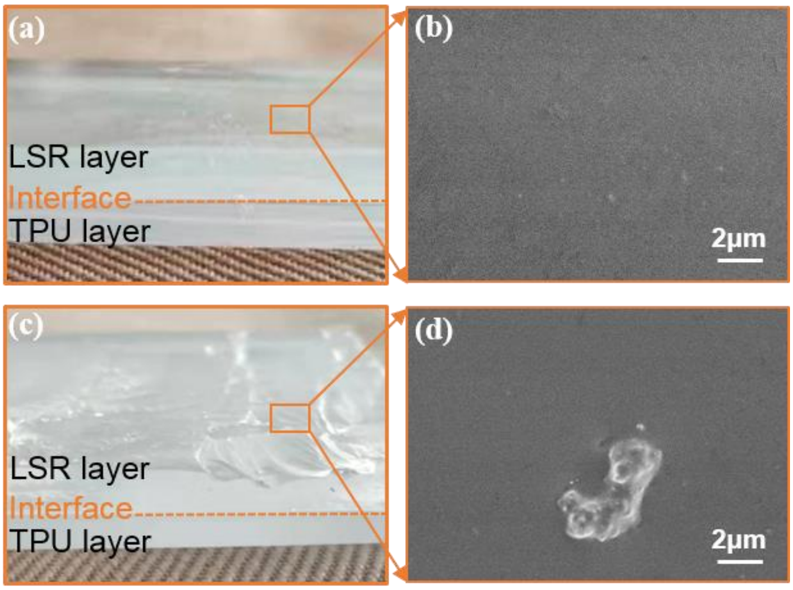

Figure S3. (a) The optical photograph and (b) SEM surface morphology at the breaking area of the LSR/TPU sample with adhesive failure, (c) the optical photograph and (d) SEM surface morphology at the breaking area of the SA-LSR/TPU sample (AP-2 content of 2 wt.%) with cohesive failure.
